# Diagnostic Value of CRP and Serum WBC Count during Septic Two-Stage Revision of Total Hip Arthroplasties

**DOI:** 10.3390/antibiotics11081098

**Published:** 2022-08-12

**Authors:** Moritz Mederake, Ulf K. Hofmann, Sebastian Benda, Philipp Schuster, Bernd Fink

**Affiliations:** 1 Department of Trauma and Reconstructive Surgery, BG Klinik, University of Tübingen, Schnarrenbergstraße 95, 72076 Tübingen, Germany; 2 Department of Orthopedic, Trauma and Reconstructive Surgery, University of Aachen Medical Center, Pauwelsstraße 30, 52074 Aachen, Germany; 3 Department of Arthroplasty and Revision Arthroplasty, Orthopaedic Clinic Markgröningen GmbH, Kurt-Lindemann-Weg 10, 71706 Markgröningen, Germany; 4 Department of Orthopaedics and Traumatology, Paracelsus Medical Private University, Clinic Nuremberg, Prof. Ernst Nathan Straße 1, 90419 Nürnberg, Germany; 5 Orthopaedic Department, University-Hospital Hamburg-Eppendorf, Martinistrasse 52, 20246 Hamburg, Germany

**Keywords:** bone and joint infections, CRP, hip arthroplasty, infection parameters, orthopedic infections, periprosthetic joint infection, two-stage revision, white blood cell count

## Abstract

The diagnostic value of C-reactive protein (CRP) and the serum white blood cell (WBC) count is still barely defined for decision making during septic two-stage revision surgery of hip arthroplasty. We, therefore, compared these values between stages as well as between the groups without and with reinfection in 117 patients. A total of 106 patients were reinfection-free (91%). The median follow-up was 51 months. With a ΔCRP of −10 mg/L and a ΔWBC count of −1000/µL, a significant decrease between stages (*p* = 0.001) could be observed. When comparing the CRP and WBC count values between groups, however, no significant difference was found at stage one, stage two and even the difference between these two time points (reinfection-free ΔCRP of −11 mg/L and ΔWBC count of −1000/µL vs. reinfection ΔCRP of −5 mg/L (*p* = 0.131) and ΔWBC count of −1100/µL) (*p* = 0.424). The diagnostic value was poor for the calculated parameters (area under the curve (AUC) 0.5–0.6). The courses of the mean CRP values of both groups were similar. We conclude that the CRP and WBC count are not helpful to guide the decision making in individual cases.

## 1. Introduction

The number of total joint arthroplasties over the world keeps increasing and with it the number of accompanying complications [1]. While there have been advances and improvements in specific types of complications such as abrasion and wear in recent years [2] periprosthetic joint infections (PJIs) continue to represent one of the greatest challenges of total joint arthroplasty. A PJI of a total hip arthroplasty (THA) is a serious complication with an incidence of around 1–2%. In the case of a late infection (later than 4 weeks after surgery), the most common therapeutic concept is the two-stage septic revision [3,4,5]. In the first stage, all foreign material is removed followed by radical debridement. Usually, a cement spacer loaded with antibiotic is implanted. In the following interim phase, the patient is treated first with intravenously and then orally administered antibiotics according to the antibiotic susceptibility profile of the microorganisms detected. The final step is to remove the spacer, to perform another radical debridement and to implant the new prosthesis. Success rates of 90–100% could be achieved with this concept [6,7,8,9,10].

Usually the interim phase takes 6 to 12 weeks [4]. The optimal duration of this phase and the proper timing of reimplantation is crucial for the success of therapy. Therefore, a combination of the clinical status of the patient and several serum and synovial indicators are used for decision making, whether the infection is under control or still active. Despite their routine use in such cases, neither the erythrocyte sedimentation rate (ESR) nor C-reactive protein (CRP) showed good diagnostic power in previous studies for TKA. While specificity was moderate to acceptable (62–85% for ESR and 89–94% for CRP), the sensitivity was 67% or even lower. Therefore, these parameters failed to be predictive [11,12]. Similar results were reported in analyses derived from mixed collectives with TKA and THA revisions. Of note, these results were also obtained when specifically looking at the development of the CRP and ESR value between stages [13,14]. Besides CRP and ESR, Jiang et al. also evaluated the Interleukin-6 (IL-6) and Fibrinogen levels. The receiver operating characteristic (ROC) curves with areas under the curve (AUC) around 0.5–0.6 showed, however, poor diagnostic value as well [14]. One mixed collective study investigating the diagnostic value for the serum WBC count prior to stage two even only described a sensitivity of 9% and a specificity of 81% which can be classified as inadequate [15]. As bigger collectives are able to produce stronger statistical statements, mixing TKA and THA collectives appears helpful at first sight. However, we believe that different joints need to be analyzed separately for reasons such as the different biomechanical properties, characteristic microbiological spectrum in case of infection and biologic properties. To the best of our knowledge, there is only one study considering inflammatory parameters in two-stage revision exclusively of THA. In this study, Shukla et al. observed a good response of the CRP and ESR values to therapy. The noted changes were, however, not helpful in terms of their diagnostic value [16]. We, therefore, conducted a study specifically investigating the diagnostic value of the serum CRP and serum WBC count in a large cohort of patients with a two-stage septic hip revision. These two parameters are commonly used inflammatory serum markers available in every medical laboratory and they are also partly used as minor criteria of the Musculoskeletal Infection Society (MSIS) [17]. Because the two-stage revision surgery is a long-term therapy, we did not only investigate two designated time points but also the differences and the complete course between stages.

The aim of the present study was to investigate how the CRP and WBC count responded to therapy with stage-one surgery and its following anti-infective treatment. We also wanted to analyze whether there is a difference in the development of these values in cases without and with reinfection. The predictive value of the CRP and WBC count were evaluated, and we attempted to calculate the optimal thresholds. 

## 2. Materials and Methods

### 2.1. Patients and Research Workflow

The study was conducted according to the local ethical standards and the principles of the 1964 Helsinki Declaration and its later amendments. Informed consent was obtained from all subjects involved in the study. The study was approved by the local ethics board of the University Hospital of Tübingen (registration number 418/2021BO2). 

All patients who received two-stage revision surgery with confirmed PJI of their THA between 2013 and 2019 in the Orthopaedic Clinic Markgröningen were reviewed. Periprosthetic joint infection was diagnosed preoperatively in all cases according to the criteria of the Musculoskeletal Infection Society (MSIS) and the International Consensus on Musculoskeletal Infection (ICM) 2018 [17,18]. Patients with an inflammatory disease (rheumatic disorders, chronic inflammatory bowel disease, vasculitis), a follow-up with less than 24 months and missing laboratory values were excluded. The data were collected prospectively, and the medical records of all patients were reviewed for age, sex, body mass index (BMI), American Society of Anesthesiologists (ASA) score, prior revision surgeries, type of explanted prosthesis, comorbidities, laboratory parameters, follow-up and outcome (later reinfection or no reinfection). The groups no reinfection and reinfection were subdivided and then compared according to the predetermined objectives. Statistical analyses were performed as described in Section 2.4.

### 2.2. Treatment Protocol

To assess the MSIS and ICM criteria, preoperative aspiration and/or biopsy examination of the hip joint was performed before any revision was carried out. Once a PJI of THA was confirmed, stage-one surgery was performed, including the explantation of the infected prosthesis with a following radical debridement. Bacteriological and histological examination were repeated intraoperatively at stage-one surgery. Thereafter, the spacer components were implanted with a bone cement containing an individualized and specific mixture of anti-infective substances according to the antibiotic susceptibility profile of the microorganisms detected, as previously described [4,8,19,20,21]. The following anti-infective therapy was administered individually according to a microbiologist’s suggestion. After two weeks of parenteral antibiotic therapy, the antibiotic treatment was changed to oral administration for at least four more weeks. After at least 6 weeks of anti-infective therapy, the stage-two operation followed with explantation of the spacer components, another radical debridement and reimplantation of the new prosthesis. There was no regular drug holiday between stages. After stage-two surgery, anti-infective treatment followed the same protocol as after stage-one surgery for another 6 weeks postoperatively.

Follow-up examinations took place at regular time intervals for at least two years. Classification as free of reinfection was made according to Diaz-Ledezma et al. [22] if the patient met the following criteria: free from mortality related to PJI, free from subsequent surgical intervention for PJI and microbiological and clinical absence of the infection for at least 2 years. In case of suspicion of a reinfection at any time of follow-up, the MSIS criteria 2014 and the ICM criteria 2018 were applied again.

### 2.3. Laboratory Parameters

WBC count (/µL) was measured with a fully automated hematology analyzer (UniCel DxH 800; Beckman Coulter, Pasadena, CA, USA), which identifies cells based on the principle of impedance technology and light scatter. CRP (mg/L) was measured by a particle-enhanced turbidimetric immunoassay (Cobas C303; Roche, Basel, Switzerland). Both methods were performed according to the manufacturers´ recommendations. The values of both parameters were recorded at two defined time points: prior to stage-one and prior to stage-two revision surgery. Furthermore, CRP level was obtained from all patients at the first postoperative day. Further, CRP levels were obtained on a regular basis postoperatively with at least six postoperative CRP measurements. The last used CRP value was obtained between day 14 and 21. In-house thresholds—in order to be assessed as positive with regard to an infection—were set at ≥10 mg/L for CRP and >9000/µL for WBC count, respectively. Not-elevated values for CRP are simply displayed as <5 mg/L by the Cobas C303 immunoassay. To statistically evaluate cases without elevated values for CRP, their value was virtually set at 0 mg/L. Changes in those inflammatory markers were named as “ΔCRP” and “ΔWBC count” and calculated using values prior to stage one minus values prior to stage-two surgery.

### 2.4. Statistical Analyses

Statistical analyses were conducted using IBM SPSS Version 24 (IBM Corp., Armonk, NY, USA) and Microsoft Excel (Microsoft, Redmond, WA, USA). Distributions of variables within the groups were assessed by histograms and Shapiro–Wilk Test. Continuous variables are presented as medians (range) or means (standard deviation) as appropriate and categorical variables as frequencies. Comparisons were performed by Mann–Whitney U-test and Wilcoxon test as appropriate. All reported *p*-values are two-sided, with an alpha level of 0.05, and have not been adjusted for multiple testing. To determine the effect size of statistical tests, r was calculated. Values of r < 0.3, r = 0.3–0.5 and r > 0.5 were rated as small, moderate and strong effect sizes, respectively.

Receiver operating characteristic (ROC) analyses and curves were performed and generated to determine the diagnostic value of the diagnostic tests regarding reinfection. The area under the ROC curve (AUC) was calculated as a measure of diagnostic effectiveness and classified as follows: AUC < 0.6 fail, AUC 0.6–0.69 poor, AUC 0.7–0.79 fair, 0.8–0.89 good, AUC 0.9–1 excellent [23]. To calculate the optimal threshold value of the laboratory tests, Youden´s J-statistics were performed. 

## 3. Results

### 3.1. Collective

Our collective consisted of 117 patients with a septic two-stage revision surgery of their THA. The median age was 71 (27–92) years, with 48 women and 69 men (41:59%). The mean BMI was 29.3 ± 6.06 kg/m^2^. Diabetes mellitus was known in 20 patients (17%) and the ASA score was as follows: ASA 1:2 patients, ASA 2: 53 patients, ASA 3: 61 patients and ASA 4: 1 patient. Explanted prostheses were in 68% of the primary and in 32% of the revision implants. The median follow-up was 51 (24–87) months. A total of 106 patients (91%) remained without reinfection during the follow-up. All the patients with a reinfection were either ASA 2 (6%) or ASA 3 (13%). Of note, patients with obesity (BMI > 30 kg/m^2^) had a 15% reinfection rate versus 6% in patients with a BMI under 30 kg/m^2^.

### 3.2. Treatment Response

The median CRP decreased in the whole collective from the prior stage-one to the prior stage-two surgery from 16 mg/L (0–357) to 8 mg/L (0–89), having a median ΔCRP of −10 mg/L (−338–79) (*p* = 0.001). With a median ΔWBC count of −1000/µL (−9000–6000), being reduced from 7000/µL (4000–16,000) to 6000/µL (3000–13,000) after treatment, the WBC count also decreased significantly in the whole collective (*p* = 0.001)

**Figure 1:**
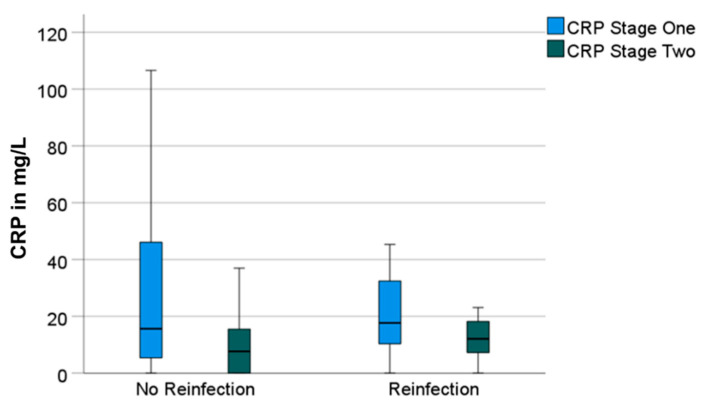
Boxplots for C-reactive protein (“CRP“) values prior to stage-one and prior to stage-two surgeries, divided in groups without and with reinfection.

**Figure 2:**
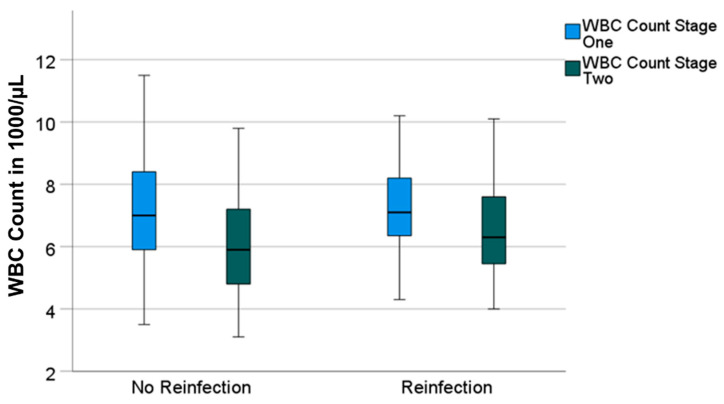
Boxplots for white blood cell count (“WBC count“) values prior to stage-one and prior to stage-two surgeries, divided in groups without and with reinfection.

Having a look at the reinfection-free group, the median CRP and WBC count also decreased significantly from 16 mg/L (0–357) to 8 mg/L (0–89) (*p* = 0.001) and from 7000/µL (3500–16,200) to 5900/µL (3100–12,300) (*p* = 0.001), respectively. Both parameters also decreased in the reinfection group—however, not significantly. The median CRP decreased from 18 mg/L (0–115) to 12 mg/L (0–41) (*p* = 0.131) and the median WBC count decreased from 7100/µL (4300–11,000) to 6300/µL (4000–12,500) (*p* = 0.424) (Figure 1 and Figure 2). 

Having a big difference in the group size (106 vs. 11 patients), the effect size r for the calculation of the significance of the decrease in the values between stages one and two was determined. With r = 0.34 in the group without reinfection and r = 0.32 in the group with reinfection, the effect size of the difference in the CRP can be classified as moderate in both groups. With r = 0.43 and r = 0.17, the effect size of the difference in the WBC count in the group without reinfection can be classified as moderate as well. The effect size in the group with reinfection must be classified as small.

### 3.3. Diagnostic Value and Determination of Optimal Threshold

Regarding the differences between the reinfection-free and the reinfection group, no significant difference could be seen neither for the CRP nor for the WBC count in both stages. Furthermore, there was also no significant difference in the ΔCRP and ΔWBC count (Table 1). 

**Figure 3:**
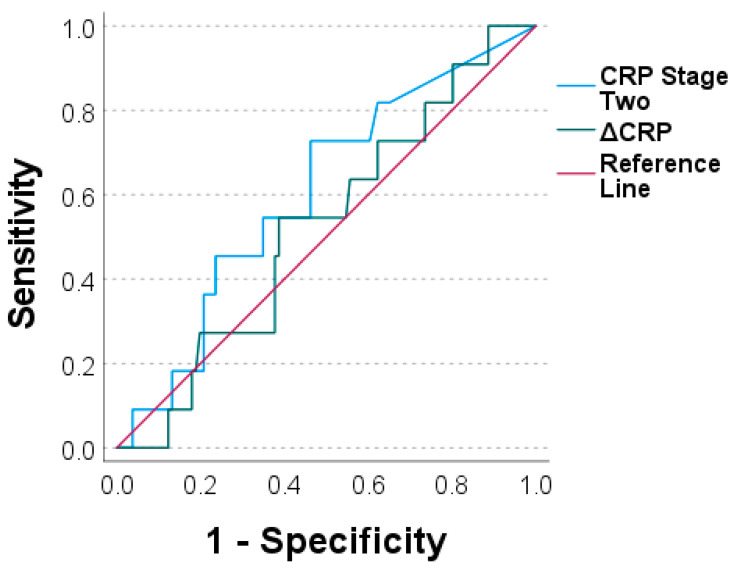
Receiver operating characteristic curves for C-reactive protein prior to stage two (“CRP stage two“) and for ΔCRP (CRP stage two minus CRP stage one).

**Figure 4:**
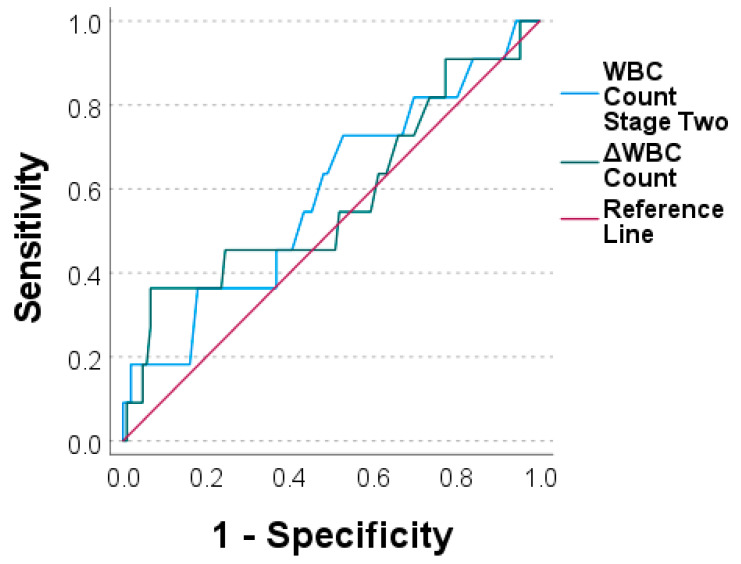
Receiver operating characteristic curves for white blood cell count prior to stage two (“WBC count stage two“) and for ΔWBC count (WBC count stage two minus WBC count stage one).

To assess the diagnostic value of both markers, we performed ROC analyses and generated ROC curves. The values for the CRP prior stage-two surgery and the ΔCRP were an AUC = 0.604 and an AUC = 0.524, respectively (Figure 3). Similar values were calculated for the WBC count for prior stage-two surgery with an AUC = 0.586 and for the ΔWBC count with an AUC = 0.579 (Figure 4).

The optimal threshold values were calculated with Youden´s J. The sensitivity and specificity were measured. However, neither the CRP or the WBC count at stage two nor the differences between the stages reached an adequate diagnostic value (Table 2).

### 3.4. CRP Course in the Interim Phase

**Figure 5:**
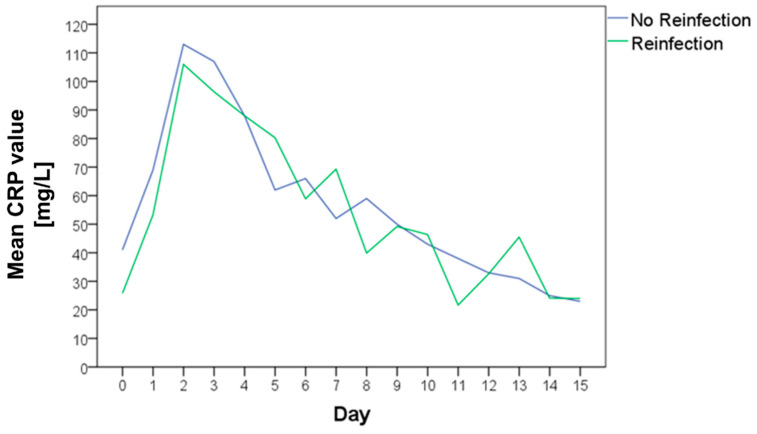
Mean C-reactive protein (CRP) value preoperatively and in the further clinical course of patients without reinfection (blue curve) and with reinfection (green curve).

**Figure 6:**
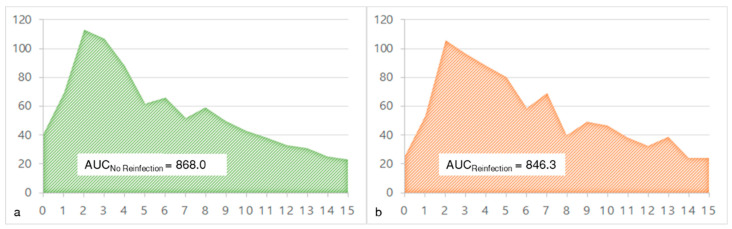
The area under the curve (AUC) of the mean C-reactive protein (CRP) values preoperatively and in the further clinical course of cases without reinfection ((**a**) green curve) and with reinfection ((**b**) orange curve).

The CRP was measured preoperatively and at multiple time points postoperatively. There was no statistically significant difference between the CRP level of the cases with or without reinfection neither preoperatively nor at any point in the follow-up (Table 3). The mean CRP values of both groups are shown in Figure 5. The area under the curve was nearly identical with 868.0 for cases without reinfection and 846.3 for cases with reinfection (Figure 6).

## 4. Discussion

Septic two-stage revision surgery is a standard treatment for a PJI of a THA with success rates of up to 90–100% [6,7,8,9,10]. It is, however, extremely important to filter out subsequent therapy failures at an early stage in order to be able to adapt the therapy. This requires parameters that guide the decision as to whether an infection is controlled or not. A lot of studies are available on the diagnostic workup prior to septic revision surgery, leading to standardized preoperative decision making according to the MSIS and ICM criteria [17,18]. However, there is an absence of such a standardized preoperative diagnostic workup to guide the timing of stage-two surgery and the decision as to whether a PJI is controlled or not. Those studies available for the diagnostic workup prior to stage two mainly report on TKA or mixed collectives with only one single study for a THA collective [11,12,13,14,16]. Because we believe that joint-specific diagnostic and therapeutic algorithms are necessary, there is a need for studies investigating this subject exclusively for THA. 

We, therefore, conducted a study regarding the CRP and WBC count. These parameters are part of the routinely obtained preoperative laboratory sample and are still used to evaluate infections by many surgeons. 

Our representative collective consisted of 117 patients and a reinfection-free rate of 91%. In line with the literature, patients with a higher ASA score and patients with obesity tended to have a higher risk for reinfection [24,25,26].

To answer our first objective, we compared the CRP and WBC count values prior to stage one with values prior to stage two. Both parameters decreased significantly between the stages (*p* = 0.001), showing the anti-infective impact of the therapy. This is in line with the high success rate of 91% in the present collective at a minimum follow-up of two years. Shukla et al. also found a significant decrease for the ESR, CRP and WBC count of the synovial fluid, which is in line with our results [16]. This also seems to be true for TKA [12].

For our second objective, we compared the values of the reinfection-free with the reinfection group. The CRP and WBC count decreased significantly in the reinfection-free group (*p* = 0.001). This difference was not significant between the stages in the reinfection group. There is also one comparative study with a TKA collective showing a similar observation [12]. These results have to be handled with caution, however, because of the different group sizes and the corresponding small effect sizes of the statistical tests. When comparing the deltas of both groups, we were not able to find a significant difference. Having a look at the literature, similar results could be obtained for THA as well as for mixed collectives for both absolute and relative differences [13,14,16]. 

To evaluate the diagnostic value, we calculated the AUC of the ROC curves. With an AUC between 0.5 and 0.6, we obtained similar results as other comparable studies [13,14,16]. Taken together, this classifies the CRP and WBC count prior to stage two as well as the ΔCRP and ΔWBC count as poor diagnostic means which are not able to discriminate between cases with reinfection and those with no reinfection.

In an attempt to define suitable thresholds, we performed Youden´s J-statistics on the data set. Yet, even with calculated optimized thresholds, we were not able to generate adequate sensitivity or specificity. This is in line with the results of Shukla et al. [16]. The CRP and WBC count are therefore not good enough to be used for decision making alone.

To answer our fifth and last objective, we compared the course of the CRP between both stages. There was no statistically significant difference in the CRP value at any time point and the areas under the curves were similar for both groups. Therefore, in our opinion, the CRP course is also not suitable for predicting the success or failure of the therapy. There is only one comparative study investigating the course of the CRP value. Interestingly, they were able to find significantly higher median prereimplantation CRP levels for the reinfection group. Furthermore, they classified the CRP trends somewhat arbitrarily and found that cases with a CRP fluctuation without dropping under 10 mg/dl are more likely to fail in therapy [27]. 

Taken together, no single CRP value is suitable to predict the success or failure of the therapy. Single values appear too sensitive for disturbance variables. Further research will show whether the course of CRP in the interim phase may be helpful to guide the therapy.

Besides the CRP and WBC count which were evaluated in the present study, there are more parameters in the literature which have been investigated: ESR, IL-6 or Fibrinogen levels in serum, for example, proved also of little help to indicate persistent infection [12,13,14]. Regarding the WBC count in the synovial fluid, various studies reported inconsistent results. While Kusuma et al., for example, found an elevated WBC in the synovial fluid not indicative of a persistent PJI in a TKA, Shukla et al. were able to show good sensitivity and excellent specificity in THA [12,16].

What always has to be kept in mind when interpreting inflammation parameters is that not only the obvious PJI is able to influence those values but also other confounders. In the case of infections of the upper respiratory tract or the urinary tract as well as after sports or trauma, inflammation parameters can also increase significantly although not always clinically obvious [28,29,30,31]. Obesity also leads to higher inflammation markers, which is one more very common reason for possibly confounding the results [32].

The strengths of our study are the consistent therapy regimen with the individualized anti-infective therapy and the course of the CRP value in the interim phase. Furthermore, the extended follow-up with at least two years should be long enough to be able to identify late periprosthetic reinfections [22]. What is more, we tried to exclude possible confounders of the inflammatory parameters by excluding chronic inflammatory diseases.

Although we have one of the biggest study collectives when compared to other studies, one clear limitation of the study collective is the small group of patients with reinfection, limiting the effect size of the statistical tests. However, this is a well-known problem which other study collectives also had to face [11,12,14,16]. Another weakness is that the CRP was not further specified when negative. This could distort the results and make the calculation of relative changes impossible. However, such relative changes were also found with little diagnostic value in the literature [13,14].

## 5. Conclusions

In this study, we were able to define the predictive values for the CRP and WBC count in one of the largest THA-only collectives. Furthermore, we did not only compare two time points but also the course of the CRP value and the AUC. Taken together, neither the inflammation parameters prior to stage one or two nor their calculated differences are helpful to guide the decision making in an individual case whether a PJI is controlled or not. There are no thresholds with good sensitivity and specificity. For this reason, these markers seem inadequate diagnostic tests for the prediction of success. Following the postoperative course of CRP in the interim phase is also of little predictive value, because there is no difference in the cases without and with reinfection. Of note, there are some frequent confounders for serum inflammatory parameters, which make the interpretation more difficult. Nevertheless, serum markers will still have their role in therapy monitoring, but they should not be used as single diagnostic tools. As a result of this study, we changed our own diagnostic algorithm by performing a prereimplantation joint aspiration in every single case and put more emphasis on the synovial fluid WBC count and the percentage of the polymorphonuclear cells. Furthermore, we are planning an investigation of the predictive value of these synovial fluid parameters; however, we are not able to provide sufficient data yet. 

Determining the potential success of a two-stage revision procedure will continue to be based on complex algorithms including a multitude of different factors. Hopefully, future research will provide new means that will simplify surgical decision making. 

## Figures and Tables

**Table 1:** Median values of CRP, WBC count, ΔCRP and ΔWBC count (CRP: C-reactive protein; WBC: white blood cell).

	Reinfection Free	Reinfection	*p*-Value
**CRP stage one in mg/L** (median/range)	16 (0–357)	18 (0–115)	0.793
**CRP stage two in mg/L** (median/range)	8 (0–89)	12 (0–41)	0.249
**ΔCRP (stage two–stage one) in mg/L** (median/range)	−11 (−338–79)	−5 (−74–14)	0.794
**WBC count stage one in 1000/µL** (median/range)	7.0 (3.5–16.2)	7.1 (4.3–11)	0.848
**WBC count stage two in 1000/µL** (median/range)	5.9 (3.1–12.3)	6.3 (4–12.5)	0.350
**ΔWBC count (stage two–stage one) in 1000/µL** (median/range)	−1.0 (−8.7–6.3)	−1.1 (−4.4–4.5)	0.390

**Table 2:** Calculated threshold values for C-reactive protein prior to stage two (“CRP stage two“), ΔCRP (CRP stage two minus CRP stage one), white blood cell count prior to stage two (“WBC count stage two“) and ΔWBC Count (WBC count stage two minus WBC count stage one).

	Calculated Threshold Value	Sensitivity	Specificity
**CRP stage two**	8.1 mg/L	0.727	0.538
**ΔCRP**	−5.1 mg/L	0.545	0.613
**WBC count stage two**	5800/µL	0.727	0.472
**ΔWBC count**	1150/µL	0.364	0.934

**Table 3:** Mean CRP values (in mg/L) preoperatively and at different points of follow-up.

	No Reinfection	Reinfection	*p*-Value
**Preoperatively**	41 ± 64	26 ± 34	0.913
Day 1	69 ± 57	53 ± 32	0.520
Day 2	114 ± 67	106 ± 35	0.769
Day 3	109 ± 54	96 ± 27	0.692
Day 4	88 ± 52	n.a.	-
Day 5	62 ± 39	80 ± 35	0.201
Day 6	66 ± 51	59 ± 25	0.864
Day 7	53 ± 44	69 ± 46	0.311
Day 8	58 ± 51	40 ± 30	0.706
Day 9	50 ± 36	n.a.	-
Day 10	43 ± 33	46 ± 18	0.519
Day 11	38 ± 26	38 ± 22	0.769
Day 12	33 ± 17	33 ± 23	0.930
Day 13	32 ± 20	n.a.	-
Day 14	25 ± 25	n.a.	-
Day 14–21	23 ± 24	24 ± 8	0.436

## Data Availability

Data are available from the authors on reasonable request.
